# 4-Methyl-*N*-(2-methyl­phen­yl)benzene­sulfonamide

**DOI:** 10.1107/S1600536809049411

**Published:** 2009-11-21

**Authors:** P. G. Nirmala, B. Thimme Gowda, Sabine Foro, Hartmut Fuess

**Affiliations:** a Department of Chemistry, Mangalore University, Mangalagangotri 574 199, Mangalore, India; b Institute of Materials Science, Darmstadt University of Technology, Petersenstrasse 23, D-64287 Darmstadt, Germany

## Abstract

In the title compound, C_14_H_15_NO_2_S, the dihedral angle between the aromatic rings is 49.7 (1)°. In the crystal, inversion dimers linked by pairs of N—H⋯O hydrogen bonds occur.

## Related literature

For our study of the effect of substituents on the crystal structures of *N*-(ar­yl)-aryl­sulfonamides, see: Gowda *et al.* (2008 ▶; 2009 ▶). For bond lengths in other aryl sulfonamides, see: Gelbrich *et al.* (2007 ▶); Perlovich *et al.* (2006 ▶).
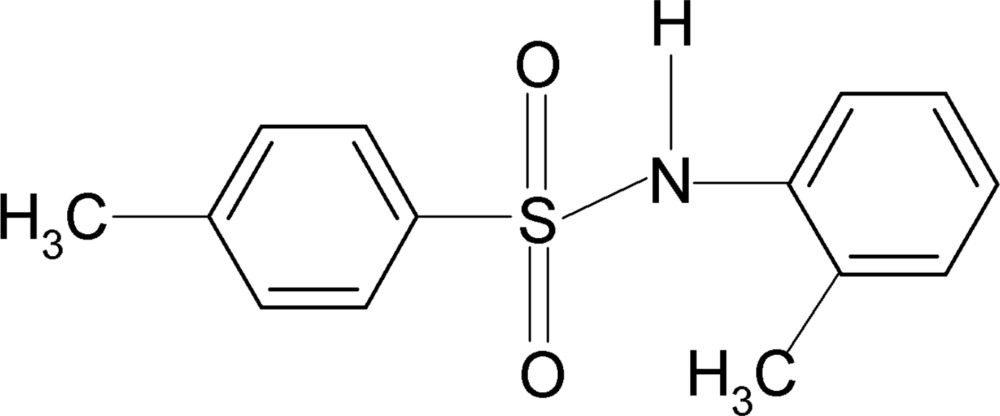


## Experimental

### 

#### Crystal data

C_14_H_15_NO_2_S
*M* *_r_* = 261.33
Orthorhombic, 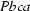

*a* = 14.650 (3) Å
*b* = 12.019 (1) Å
*c* = 15.634 (3) Å
*V* = 2752.8 (8) Å^3^
*Z* = 8
Cu *K*α radiation
μ = 2.04 mm^−1^
*T* = 299 K
0.55 × 0.55 × 0.35 mm

#### Data collection

Enraf–Nonius CAD-4 diffractometer
Absorption correction: ψ scan (North *et al.*, 1968 ▶) *T*_min_ = 0.400, *T*_max_ = 0.535
3596 measured reflections
2450 independent reflections
1899 reflections with *I* > 2σ(*I*)
*R*_int_ = 0.044
3 standard reflections frequency: 120 min intensity decay: 1.0%

#### Refinement

*R*[*F* ^2^ > 2σ(*F* ^2^)] = 0.044
*wR*(*F* ^2^) = 0.134
*S* = 1.06
2450 reflections
167 parameters
1 restraint
H atoms treated by a mixture of independent and constrained refinement
Δρ_max_ = 0.18 e Å^−3^
Δρ_min_ = −0.23 e Å^−3^

### 

Data collection: *CAD-4-PC* (Enraf–Nonius, 1996 ▶); cell refinement: *CAD-4-PC*; data reduction: *REDU4* (Stoe & Cie, 1987 ▶); program(s) used to solve structure: *SHELXS97* (Sheldrick, 2008 ▶); program(s) used to refine structure: *SHELXL97* (Sheldrick, 2008 ▶); molecular graphics: *PLATON* (Spek, 2009 ▶); software used to prepare material for publication: *SHELXL97* .

## Supplementary Material

Crystal structure: contains datablocks I, global. DOI: 10.1107/S1600536809049411/ng2688sup1.cif

Structure factors: contains datablocks I. DOI: 10.1107/S1600536809049411/ng2688Isup2.hkl

Additional supplementary materials:  crystallographic information; 3D view; checkCIF report

## Figures and Tables

**Table 1:** Hydrogen-bond geometry (Å, °)

*D*—H⋯*A*	*D*—H	H⋯*A*	*D*⋯*A*	*D*—H⋯*A*
N1—H1*N*⋯O1^i^	0.838 (17)	2.219 (18)	3.036 (3)	165 (2)

Symmetry code: (i) 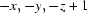.

**Fig. 1.**
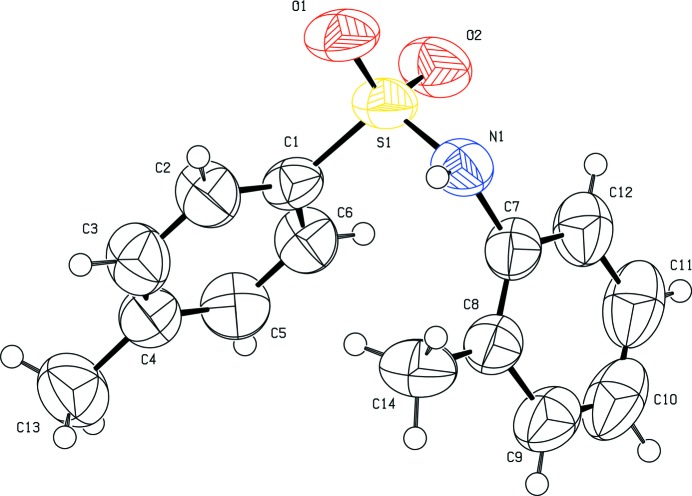
Molecular structure of (I), showing the atom labelling scheme and displacement ellipsoids are drawn at the 50% probability level.

**Fig. 2.**
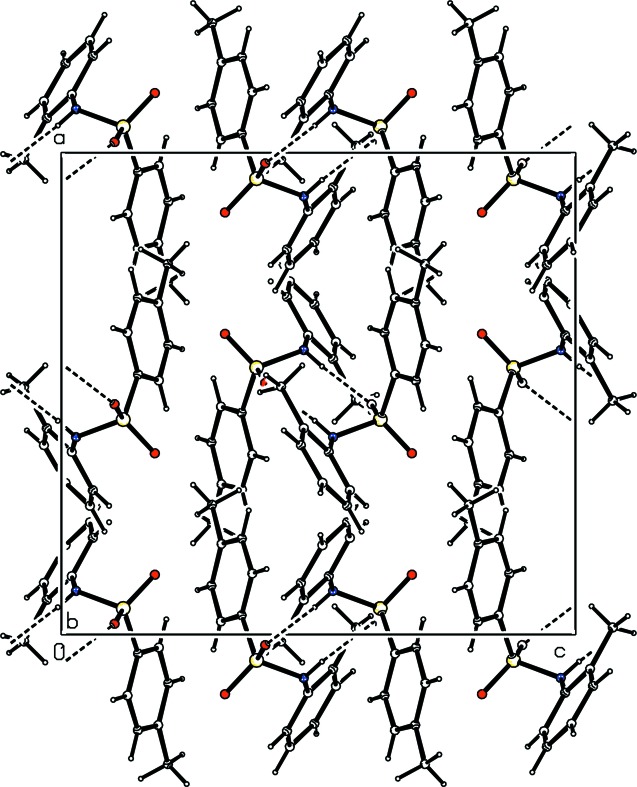
Molecular packing of (I) with hydrogen bonding shown as dashed lines.

## supplementary crystallographic information

### Comment

As part of a study of the effect of substituents on the crystal structures of *N*-(aryl)-arylsulfonamides (Gowda *et al.*, 2008; 2009), in the present work, the structure of 4-methyl-*N*-(2-methylphenyl)- benzenesulfonamide (I) has been determined. The conformation of the N—C bond in the C1—SO_2_—NH—C7 segment of the structure has *gauche* torsions with respect to the SO bonds.(Fig. 1). The molecule is bent at the S atom with the C1—SO_2_—NH—C7 torsion angle of 60.0 (2)°, compared to the values of 72.0 (2)° in *N*-(2-methylphenyl)- benzenesulfonamide (II)(Gowda *et al.*,2008) and 51.6 (3)° in 4-methyl-*N*-(phenyl)benzenesulfonamide (III) (Gowda *et al.*, 2009). The N—H bond orients itself away from the *ortho*-methyl group in the anilino benzene ring. The two benzene rings in (I) are tilted relative to each other by 49.7 (1)°, compared to the values of 61.5 (1)° in (II) and 68.4 (1)° in (III). The other bond parameters are similar to those observed in (II), (III) and other aryl sulfonamides(Perlovich *et al.*, 2006; Gelbrich *et al.*, 2007). The crystal packing of molecules in (I) *via* N—H···O(S) hydrogen bonds (Table 1) is shown in Fig.2.

### Experimental

The solution of toluene (10 cc) in chloroform (40 cc) was treated dropwise with chlorosulfonic acid (25 cc) at 0 ° C. After the initial evolution of hydrogen chloride subsided, the reaction mixture was brought to room temperature and poured into crushed ice in a beaker. The chloroform layer was separated, washed with cold water and allowed to evaporate slowly. The residual benzenesulfonylchloride was treated with *o*-toluidine in the stoichiometric ratio and boiled for ten minutes. The reaction mixture was then cooled to room temperature and added to ice cold water (100 cc). The resultant solid 4-methyl-*N*-(2-methylphenyl)benzenesulfonamide was filtered under suction and washed thoroughly with cold water. It was then recrystallized to constant melting point from dilute ethanol. The purity of the compound was checked and characterized by recording its infrared and NMR spectra. The single crystals used in X-ray diffraction studies were grown in ethanolic solution by a slow evaporation at room temperature.

### Refinement

The H atom of the NH group was located in a difference map and later restrained to the distance N—H = 0.86 (2) Å. The other H atoms were positioned with idealized geometry using a riding model [C—H = 0.93–0.96 Å]. All H atoms were refined with isotropic displacement parameters (set to 1.2 times of the *U*_eq_ of the parent atom).

### Crystal data

**Table:**

C_14_H_15_NO_2_S	*F*(000) = 1104
*M**_r_* = 261.33	*D*_x_ = 1.261 Mg m^−^^3^
Orthorhombic, *P**b**c**a*	Cu *K*α radiation, λ = 1.54180 Å
Hall symbol: -P 2ac 2ab	Cell parameters from 25 reflections
*a* = 14.650 (3) Å	θ = 5.7–18.7°
*b* = 12.019 (1) Å	µ = 2.04 mm^−^^1^
*c* = 15.634 (3) Å	*T* = 299 K
*V* = 2752.8 (8) Å^3^	Prism, colourless
*Z* = 8	0.55 × 0.55 × 0.35 mm

### Data collection

**Table:**

Enraf–Nonius CAD-4 diffractometer	1899 reflections with *I* > 2σ(*I*)
Radiation source: fine-focus sealed tube	*R*_int_ = 0.044
graphite	θ_max_ = 66.8°, θ_min_ = 5.5°
ω–2θ scans	*h* = −17→1
Absorption correction: ψ scan (North *et al.*, 1968)	*k* = −14→1
*T*_min_ = 0.400, *T*_max_ = 0.535	*l* = −6→18
3596 measured reflections	3 standard reflections every 120 min
2450 independent reflections	intensity decay: 1.0%

### Refinement

**Table:**

Refinement on *F*^2^	Secondary atom site location: difference Fourier map
Least-squares matrix: full	Hydrogen site location: inferred from neighbouring sites
*R*[*F*^2^ > 2σ(*F*^2^)] = 0.044	H atoms treated by a mixture of independent and constrained refinement
*wR*(*F*^2^) = 0.134	*w* = 1/[σ^2^(*F*_o_^2^) + (0.066*P*)^2^ + 0.6125*P*] where *P* = (*F*_o_^2^ + 2*F*_c_^2^)/3
*S* = 1.06	(Δ/σ)_max_ < 0.001
2450 reflections	Δρ_max_ = 0.18 e Å^−^^3^
167 parameters	Δρ_min_ = −0.23 e Å^−^^3^
1 restraint	Extinction correction: *SHELXL97* (Sheldrick, 2008), Fc^*^=kFc[1+0.001xFc^2^λ^3^/sin(2θ)]^-1/4^
Primary atom site location: structure-invariant direct methods	Extinction coefficient: 0.0052 (4)

### Special details

**Table:**

Geometry. All e.s.d.'s (except the e.s.d. in the dihedral angle between two l.s. planes) are estimated using the full covariance matrix. The cell e.s.d.'s are taken into account individually in the estimation of e.s.d.'s in distances, angles and torsion angles; correlations between e.s.d.'s in cell parameters are only used when they are defined by crystal symmetry. An approximate (isotropic) treatment of cell e.s.d.'s is used for estimating e.s.d.'s involving l.s. planes.
Refinement. Refinement of *F*^2^ against ALL reflections. The weighted *R*-factor *wR* and goodness of fit *S* are based on *F*^2^, conventional *R*-factors *R* are based on *F*, with *F* set to zero for negative *F*^2^. The threshold expression of *F*^2^ > σ(*F*^2^) is used only for calculating *R*-factors(gt) *etc*. and is not relevant to the choice of reflections for refinement. *R*-factors based on *F*^2^ are statistically about twice as large as those based on *F*, and *R*- factors based on ALL data will be even larger.

### Fractional atomic coordinates and isotropic or equivalent isotropic displacement parameters (Å^2^)

**Table:**

	*x*	*y*	*z*	*U*_iso_*/*U*_eq_	
C1	−0.03969 (15)	0.17693 (18)	0.64937 (14)	0.0620 (6)	
C2	−0.12664 (19)	0.1429 (2)	0.6286 (2)	0.0857 (8)	
H2	−0.1359	0.0754	0.6008	0.103*	
C3	−0.19886 (19)	0.2088 (3)	0.6489 (2)	0.0898 (8)	
H3	−0.2574	0.1845	0.6356	0.108*	
C4	−0.18818 (18)	0.3102 (2)	0.68837 (15)	0.0733 (6)	
C5	−0.10123 (19)	0.3429 (2)	0.70810 (17)	0.0817 (7)	
H5	−0.0923	0.4112	0.7348	0.098*	
C6	−0.02714 (18)	0.2779 (2)	0.68964 (16)	0.0744 (7)	
H6	0.0313	0.3017	0.7041	0.089*	
C7	0.12097 (16)	0.2537 (2)	0.52015 (16)	0.0689 (6)	
C8	0.07178 (18)	0.3294 (2)	0.47154 (17)	0.0722 (7)	
C9	0.1070 (2)	0.4370 (2)	0.4659 (2)	0.0922 (9)	
H9	0.0766	0.4895	0.4328	0.111*	
C10	0.1853 (3)	0.4672 (3)	0.5079 (3)	0.1076 (11)	
H10	0.2063	0.5400	0.5040	0.129*	
C11	0.2329 (2)	0.3912 (3)	0.5557 (2)	0.1046 (11)	
H11	0.2862	0.4118	0.5839	0.125*	
C12	0.20070 (18)	0.2843 (2)	0.5612 (2)	0.0871 (8)	
H12	0.2329	0.2318	0.5928	0.105*	
C13	−0.2691 (2)	0.3820 (3)	0.7071 (2)	0.1058 (10)	
H13A	−0.3106	0.3425	0.7437	0.127*	
H13B	−0.2993	0.4008	0.6546	0.127*	
H13C	−0.2493	0.4489	0.7352	0.127*	
C14	−0.0145 (2)	0.2995 (2)	0.4273 (2)	0.0950 (9)	
H14A	−0.0583	0.2740	0.4686	0.114*	
H14B	−0.0028	0.2415	0.3865	0.114*	
H14C	−0.0381	0.3637	0.3983	0.114*	
N1	0.09033 (14)	0.14056 (16)	0.52843 (14)	0.0700 (5)	
H1N	0.0544 (16)	0.118 (2)	0.4904 (15)	0.084*	
O1	0.02213 (13)	−0.01564 (13)	0.60343 (13)	0.0853 (6)	
O2	0.12395 (13)	0.11202 (15)	0.68176 (13)	0.0885 (6)	
S1	0.05391 (4)	0.09508 (5)	0.62032 (4)	0.0692 (3)	

### Atomic displacement parameters (Å^2^)

**Table:**

	*U*^11^	*U*^22^	*U*^33^	*U*^12^	*U*^13^	*U*^23^
C1	0.0703 (13)	0.0502 (11)	0.0654 (12)	−0.0088 (10)	−0.0110 (11)	0.0036 (9)
C2	0.0693 (15)	0.0638 (14)	0.124 (2)	−0.0106 (13)	−0.0150 (15)	−0.0180 (15)
C3	0.0678 (15)	0.0908 (19)	0.111 (2)	−0.0118 (15)	−0.0145 (15)	−0.0120 (16)
C4	0.0772 (15)	0.0814 (16)	0.0614 (13)	0.0030 (13)	0.0008 (12)	0.0042 (12)
C5	0.0939 (19)	0.0689 (15)	0.0821 (16)	−0.0029 (14)	−0.0014 (15)	−0.0193 (13)
C6	0.0727 (14)	0.0705 (15)	0.0801 (15)	−0.0096 (12)	−0.0102 (13)	−0.0162 (12)
C7	0.0686 (13)	0.0572 (12)	0.0809 (15)	−0.0060 (11)	0.0124 (12)	−0.0126 (11)
C8	0.0782 (15)	0.0539 (12)	0.0844 (16)	−0.0001 (11)	0.0177 (13)	−0.0083 (11)
C9	0.111 (2)	0.0598 (14)	0.106 (2)	−0.0032 (15)	0.0331 (19)	−0.0051 (14)
C10	0.121 (3)	0.0745 (19)	0.127 (3)	−0.034 (2)	0.046 (2)	−0.025 (2)
C11	0.094 (2)	0.105 (2)	0.115 (2)	−0.038 (2)	0.019 (2)	−0.030 (2)
C12	0.0727 (15)	0.0891 (19)	0.0996 (19)	−0.0145 (14)	0.0054 (15)	−0.0142 (15)
C13	0.098 (2)	0.120 (3)	0.099 (2)	0.0238 (19)	0.0124 (18)	−0.0002 (19)
C14	0.110 (2)	0.0680 (16)	0.107 (2)	0.0131 (16)	−0.0100 (19)	0.0042 (15)
N1	0.0709 (12)	0.0544 (10)	0.0849 (14)	0.0011 (9)	−0.0057 (10)	−0.0087 (9)
O1	0.1035 (13)	0.0437 (8)	0.1087 (13)	−0.0029 (9)	−0.0176 (11)	0.0040 (8)
O2	0.0823 (11)	0.0824 (12)	0.1009 (13)	0.0038 (9)	−0.0332 (11)	0.0064 (10)
S1	0.0729 (4)	0.0495 (3)	0.0852 (4)	0.0014 (3)	−0.0171 (3)	0.0035 (3)

### Geometric parameters (Å, °)

**Table:**

C1—C2	1.377 (3)	C9—C10	1.371 (5)
C1—C6	1.380 (3)	C9—H9	0.9300
C1—S1	1.748 (2)	C10—C11	1.370 (5)
C2—C3	1.359 (4)	C10—H10	0.9300
C2—H2	0.9300	C11—C12	1.371 (4)
C3—C4	1.375 (4)	C11—H11	0.9300
C3—H3	0.9300	C12—H12	0.9300
C4—C5	1.368 (4)	C13—H13A	0.9600
C4—C13	1.495 (4)	C13—H13B	0.9600
C5—C6	1.368 (4)	C13—H13C	0.9600
C5—H5	0.9300	C14—H14A	0.9600
C6—H6	0.9300	C14—H14B	0.9600
C7—C12	1.382 (4)	C14—H14C	0.9600
C7—C8	1.387 (4)	N1—S1	1.627 (2)
C7—N1	1.437 (3)	N1—H1N	0.838 (17)
C8—C9	1.395 (4)	O1—S1	1.4344 (17)
C8—C14	1.484 (4)	O2—S1	1.4203 (18)
			
C2—C1—C6	119.5 (2)	C9—C10—H10	119.6
C2—C1—S1	119.82 (18)	C10—C11—C12	118.9 (3)
C6—C1—S1	120.62 (18)	C10—C11—H11	120.5
C3—C2—C1	119.5 (2)	C12—C11—H11	120.5
C3—C2—H2	120.3	C11—C12—C7	120.7 (3)
C1—C2—H2	120.3	C11—C12—H12	119.6
C2—C3—C4	122.2 (3)	C7—C12—H12	119.6
C2—C3—H3	118.9	C4—C13—H13A	109.5
C4—C3—H3	118.9	C4—C13—H13B	109.5
C5—C4—C3	117.5 (3)	H13A—C13—H13B	109.5
C5—C4—C13	121.9 (3)	C4—C13—H13C	109.5
C3—C4—C13	120.6 (3)	H13A—C13—H13C	109.5
C6—C5—C4	121.8 (2)	H13B—C13—H13C	109.5
C6—C5—H5	119.1	C8—C14—H14A	109.5
C4—C5—H5	119.1	C8—C14—H14B	109.5
C5—C6—C1	119.5 (2)	H14A—C14—H14B	109.5
C5—C6—H6	120.2	C8—C14—H14C	109.5
C1—C6—H6	120.2	H14A—C14—H14C	109.5
C12—C7—C8	121.2 (2)	H14B—C14—H14C	109.5
C12—C7—N1	118.3 (3)	C7—N1—S1	119.98 (16)
C8—C7—N1	120.5 (2)	C7—N1—H1N	115.9 (19)
C7—C8—C9	116.8 (3)	S1—N1—H1N	108 (2)
C7—C8—C14	122.6 (2)	O2—S1—O1	119.47 (11)
C9—C8—C14	120.6 (3)	O2—S1—N1	108.19 (12)
C10—C9—C8	121.6 (3)	O1—S1—N1	104.81 (11)
C10—C9—H9	119.2	O2—S1—C1	108.08 (11)
C8—C9—H9	119.2	O1—S1—C1	108.39 (11)
C11—C10—C9	120.7 (3)	N1—S1—C1	107.32 (10)
C11—C10—H10	119.6		
			
C6—C1—C2—C3	−0.7 (4)	C8—C9—C10—C11	1.5 (5)
S1—C1—C2—C3	−177.8 (2)	C9—C10—C11—C12	−0.3 (5)
C1—C2—C3—C4	1.3 (5)	C10—C11—C12—C7	−0.8 (5)
C2—C3—C4—C5	−0.9 (5)	C8—C7—C12—C11	0.8 (4)
C2—C3—C4—C13	178.0 (3)	N1—C7—C12—C11	−179.8 (3)
C3—C4—C5—C6	−0.1 (4)	C12—C7—N1—S1	68.5 (3)
C13—C4—C5—C6	−178.9 (3)	C8—C7—N1—S1	−112.0 (2)
C4—C5—C6—C1	0.6 (4)	C7—N1—S1—O2	−56.3 (2)
C2—C1—C6—C5	−0.2 (4)	C7—N1—S1—O1	175.17 (18)
S1—C1—C6—C5	176.9 (2)	C7—N1—S1—C1	60.1 (2)
C12—C7—C8—C9	0.3 (4)	C2—C1—S1—O2	−150.3 (2)
N1—C7—C8—C9	−179.1 (2)	C6—C1—S1—O2	32.6 (2)
C12—C7—C8—C14	−179.5 (2)	C2—C1—S1—O1	−19.5 (3)
N1—C7—C8—C14	1.1 (4)	C6—C1—S1—O1	163.5 (2)
C7—C8—C9—C10	−1.4 (4)	C2—C1—S1—N1	93.2 (2)
C14—C8—C9—C10	178.4 (3)	C6—C1—S1—N1	−83.8 (2)

### Hydrogen-bond geometry (Å, °)

**Table:**

*D*—H···*A*	*D*—H	H···*A*	*D*···*A*	*D*—H···*A*
N1—H1N···O1^i^	0.84 (2)	2.22 (2)	3.036 (3)	165 (2)

Symmetry codes: (i) −*x*, −*y*, −*z*+1.

## References

[bb1] Enraf–Nonius (1996). *CAD-4-PC*. Enraf–Nonius, Delft, The Netherlands.

[bb2] Gelbrich, T., Hursthouse, M. B. & Threlfall, T. L. (2007). *Acta Cryst.* B**63**, 621–632. 10.1107/S010876810701395X 17641433

[bb3] Gowda, B. T., Foro, S., Babitha, K. S. & Fuess, H. (2008). *Acta Cryst.* E**64**, o1692. 10.1107/S1600536808024562 PMC2960504 21201681

[bb4] Gowda, B. T., Foro, S., Nirmala, P. G., Terao, H. & Fuess, H. (2009). *Acta Cryst.* E**65**, o1219. 10.1107/S1600536809016377 PMC2969678 21583088

[bb5] North, A. C. T., Phillips, D. C. & Mathews, F. S. (1968). *Acta Cryst.* A**24**, 351–359.

[bb6] Perlovich, G. L., Tkachev, V. V., Schaper, K.-J. & Raevsky, O. A. (2006). *Acta Cryst.* E**62**, o780–o782.

[bb7] Sheldrick, G. M. (2008). *Acta Cryst.* A**64**, 112–122. 10.1107/S0108767307043930 18156677

[bb8] Spek, A. L. (2009). *Acta Cryst.* D**65**, 148–155. 10.1107/S090744490804362X PMC2631630 19171970

[bb9] Stoe & Cie (1987). *REDU4*. Stoe & Cie GmbH, Darmstadt, Germany.

